# Reprogramming of orientation columns in visual cortex: a domino effect

**DOI:** 10.1038/srep09436

**Published:** 2015-03-24

**Authors:** Lyes Bachatene, Vishal Bharmauria, Sarah Cattan, Jean Rouat, Stéphane Molotchnikoff

**Affiliations:** 1 Département de Sciences Biologiques, Université de Montréal, Montréal, QC, Canada, H3C 3J7; 2 Neurosciences Computationnelles et Traitement Intelligent des Signaux (NECOTIS, Département de Génie Électrique et Génie Informatique, Université de Sherbrooke, Sherbrooke, Québec, Canada, J1K 2R1)

## Abstract

Cortical organization rests upon the fundamental principle that neurons sharing similar properties are co-located. In the visual cortex, neurons are organized into orientation columns. In a column, most neurons respond optimally to the same axis of an oriented edge, that is, the preferred orientation. This orientation selectivity is believed to be absolute in adulthood. However, in a fully mature brain, it has been established that neurons change their selectivity following sensory experience or visual adaptation. Here, we show that after applying an adapter away from the tested cells, neurons whose receptive fields were located remotely from the adapted site also exhibit a novel selectivity in spite of the fact that they were not adapted. These results indicate a robust reconfiguration and remapping of the orientation domains with respect to each other thus removing the possibility of an orientation hole in the new hypercolumn. These data suggest that orientation columns transcend anatomy, and are almost strictly functionally dynamic.

Since the seminal investigations of Hubel and Wiesel in 1962^1^, it has been extensively established that the visual cortex is organized for orientation selectivity in a columnar fashion from pial membrane to the white matter. That is, most neurons assembled in a vertical column are selective to the same axis of orientation in response to an edge presented within their respective receptive field (RF). Indeed, if an electrode penetrates the cortex in an oblique direction, the preferred neuronal orientation rotates in a methodical fashion by about 16.5° for every traveling step of 0.09 mm of the recording tip, as it crosses from one orientation column to the next. Such systematic progression implies that, once a few reference preferred orientations of neurons are determined, it is conceivable to predict the orientation sequence. This organization rests on the notion that columnar organization is an outcome of segregated architecture of inter-neuronal connections leading to a relatively inflexible layout of striate organization. For instance, LGN neurons aligned along an axis connect to a single cortical cell^2^,^3^ giving rise to the orientation selectivity.

Yet, a few observations^4^,^5^,^6^ have reported that these columnar organizations are highly variable, even in the same species.

Interestingly, recent experiments showed that following the continuous or frequent application of non-optimal stimuli, orientation in our case (called adapter to which neurons initially responded poorly), neurons change their stimulus selectivity^7^,^8^,^9^,^10^,^11^,^12^,^13^,^14^. For orientation, depending on the duration of adaptation, neurons in area 17 shift the peak of their orientation tuning curve either in the direction of the adapter (attractive shift) or away from the adapter (repulsive shift). Whatever the direction of the shift, the newly acquired optimal orientation presents a two-fold problem. The new selectivity following adaptation may be represented twice inside the hypercolumn, and alternatively, the emergence of a novel preferred orientation creates an orientation hole since the original axis is now deleted.

A basic question then arises: do cells in neighboring columns also shift their optimal orientation even if they were not exposed to the adapting stimulus, in order to restore the uniquely fashioned regularity of orientation processing? Heuristically, this invites another question: does the entire orientation hypercolumn exhibit an altered organization? In other words, are Hubel and Wiesel columns modifiable depending on the stimulation history or, in contrast, do orientation columns retain their original orientation selectivity in spite of the fact that the neighboring column changes its orientation-preference?

Our results allow us to argue that columnar organization is a processing unit rather than an anatomically based structure. The direct implication of our described investigations is that the orientation column organization classically supported by structural connections between neurons in V1 must be reassessed. We demonstrate that original preferred orientations of neurons are changed following local adaptation executed at some distance from tested cells.

Although shifts in orientation tuning of the adapted cells were described in previous reports^9^,^10^,^11^, we were particularly interested in exploring what occurred in the non-adapted cells, i.e., if one group of cells changes its preferred orientation following adaptation, do the cells belonging to other and non-adapted orientation columns change their preferred orientation concurrently as well?

## Results

### Orientation preference and goodness of fits

**Figure 1:**
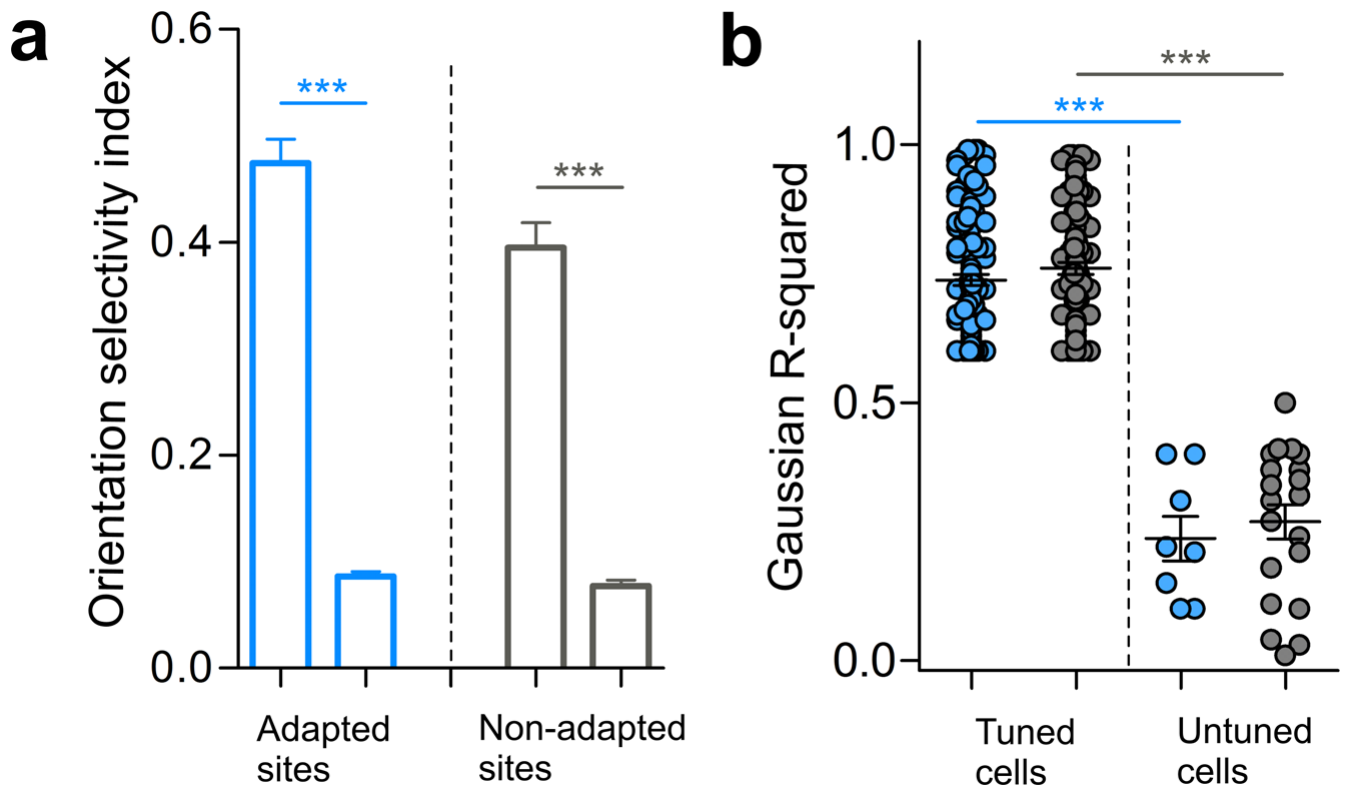
Orientation selectivity and goodness of fits. (a) Histograms showing the mean values of orientation selectivity indices (OSI's) for both sites. Significant differences lead to the classification of cells into broadly tuned cells and orientation-selective neurons. (b) Differences of the Gaussian R-squared between tuned and untuned cells in both sites (adapted sites in blue, non-adapted sites in gray).

In total, 266 cells were recorded in area 17 of anesthetized cats. Neurons were classified into two groups of cells on the basis of their orientation selectivity which was determined by the orientation selectivity index (OSI, see methods). In line with the previous reports^8^,^15^,^16^,^17^, neurons having an OSI superior or equal to 0.4 are classified as sharply tuned cells. This classification led to 218 orientation-selective neurons while 48 cells were broadly tuned. In the present investigation, tuned neurons had an OSI of 0.5 ± 0.02 and 0.4 ± 0.02 for adapted and non-adapted sites respectively, whereas broadly tuned neurons exhibited an OSI of 0.08 ± 0.004 and 0.07 ± 0.005 for adapted and non-adapted sites, respectively (Fig. 1a). In addition, we computed the Gaussian fits from the raw tuning curves to precisely compute the preferred orientation (pre- and post-adaptation) thus allowing the calculation of shift of the peak of the orientation tuning curve. For both sites, i.e., adapted and non-adapted, significant differences were observed between tuned (mean R^2^ = 0.73 ± 0.1 for adapted sites, mean R^2^ = 0.76 ± 0.1 for non-adapted sites, Fig. 1b) and untuned neurons (mean R^2^ = 0.23 ± 0.1 for adapted sites, mean R^2^ = 0.26 ± 0.1 for non-adapted sites, Fig. 1b). Due to their poor orientation selectivity, all untuned cells were excluded from further analyses.

Comparison of the orientation selectivity index for all neurons from adapted and non-adapted sites before and after adaptation was performed. The values were respectively: OSI_pre-adaptation_ = 0.44 ± 0.01 and OSI_post-adaptation_ = 0.49 ± 0.01 (n = 218). The R-squared derived from the Gaussian fits were also computed for both conditions; the R^2^_pre-adaptation_ = 0.7 ± 0.008, the R^2^_post-adaptation_ = 0.7 ± 0.01 (n = 218).

### Typical results

**Figure 2:**
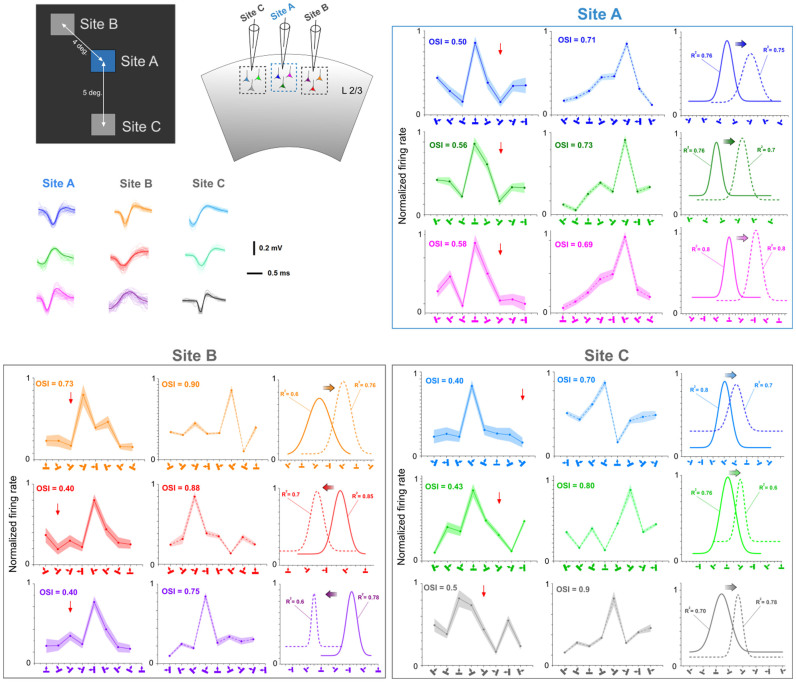
Typical examples of shifts of orientation tuning curve peaks. Tuning shifts are underlined by horizontal colored arrows. The downward red arrow heads indicate the adapting orientation in this and all figures. Upper left insert shows the respective positions of three receptive fields. Only receptive field A was adapted. Waveforms of respective sorted out action potentials are shown below the insert. Tuning curves pre- and post-adaptation are illustrated for each stimulated site (Upper right row: site A, lower left row: site B, lower right row: site C). The curves represent raw data and Gaussian fits. Light colors indicate error bars. Orientations of gratings are depicted on X-axis. OSI's and R-squared are indicated for each tuning curve.

**Figure 3:**
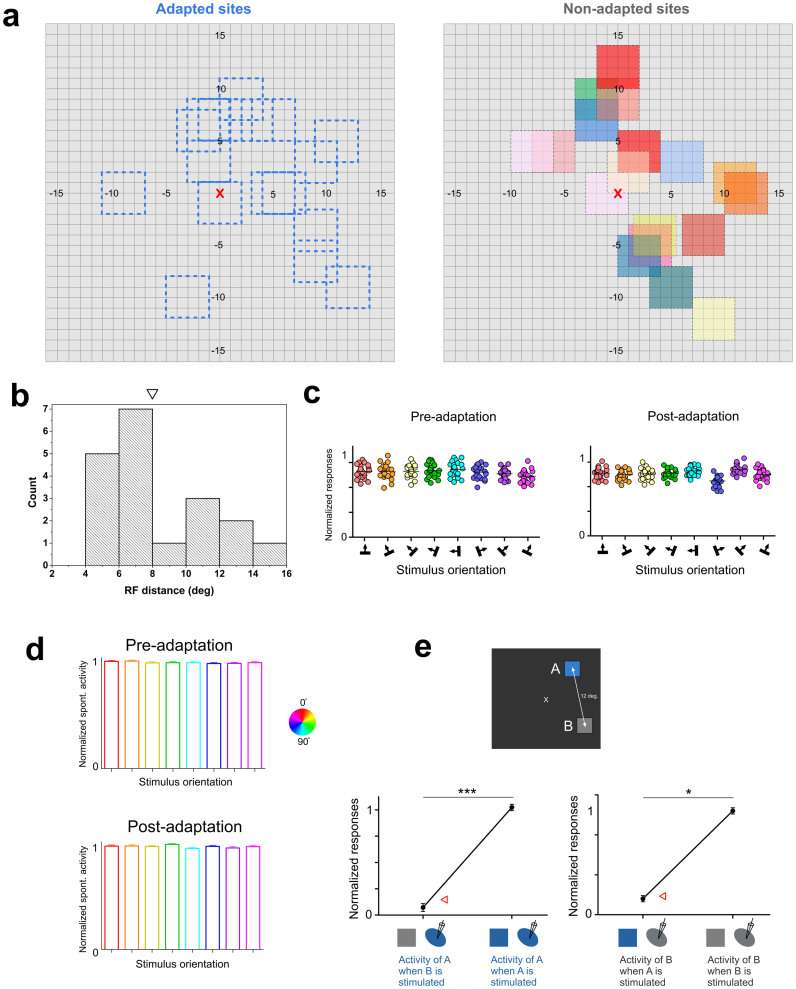
Location of receptive fields and spontaneous activity. (a) Distribution of adapted (left) and non-adapted (right) receptive fields. Color codes of non-adapted sites correspond to colors of Figure 4a. The receptive field (RF) locations are relative to the fixation point which is underlined by the red cross. (b) Histogram distribution of RF-distances between adapted and non-adapted sites. The downward triangle represents the mean value of RF distance. (c) Absence of spontaneous activity (SA) modulations of one unit when the companion cell is stimulated within its RF at tested orientations. Distance between receptive fields: 6°. The total lack of modulation of SA suggests an absence of overlap between both RF's. (d) Global results of spontaneous activity of one site while stimulating the adjacent one in both pre- and post-adaptation conditions. (e) Example of multi-unit activity of one RF during the stimulation of the companion RF and during the stimulation of the RF itself. Statistical differences were observed between these conditions. Red triangles indicate the level of spontaneous activity of the RF of interest.

As expected from previous investigations, peaks of orientation tuning curves shift following visual adaptation in anesthetized cats that were conventionally prepared for recording neurons' electrical activity. Recorded unit activity was filtered, amplified and displayed on oscilloscope and computer screens. Figure 2 shows the typical results. Three receptive fields (RF) are displayed on the top left. In this example, the center to center distance was 4° between RF's A and B and 5° between RF's A and C. Only RF A was locally adapted (see methods for details), and all three fields were stimulated one-by-one with an identical drifting sine-wave grating patch positioned at the center of each excitatory field (stimuli properties were constant). RF's B and C are positioned on opposite sides relative to the adapted area. In the first stage, multi-unit orientation tuning curves were determined from recorded cumulative activities (multiunit responses) from all three areas. Each RF was stimulated in isolation, that is, gratings were applied alternatively in each field, and it is important to emphasize that we never stimulated excitatory fields simultaneously, in order to exclude direct cross talk between groups of cells. Additionally, the spontaneous activity of unstimulated cells remained unchanged when the companion field was excited (see example in Fig. 3c).

In the second phase, the adapting grating was placed within RF A at an orientation 45° off the optimal axis, as determined in the first phase, of this particular field. Other parameters of the sine-wave patch remained unchanged. The adaptation phase lasted 12 minutes without interruption and no stimulation was applied to companion RF's that remained dark. Single units were isolated from the multiunit activity and Gaussian tuning curves were computed. The sorted waveforms at each site are displayed in the upper left part of the figure. The adaptation of RF A induced the classical shifts of the peak of the orientation tuning curves of cells belonging to this particular field. Trial-by-trial Pearson correlations of spike-counts (TC) between simultaneously recorded cells were computed in order to ascertain that cells are well isolated (Supplementary Fig. S1).

Figure 2 shows the classical attractive shifts in the adapted field for all three recorded units. This adaptation (adapting orientation indicated by downward red arrow head, in all figures; light colors in raw curves indicate error bars in all figures; Gaussian R-squared and OSI's are shown above each tuning curve) induced a roughly equal attractive displacement of the peak of the orientation tuning curves: 60.49°, 71.04°, and 96.22° for blue, green, and pink cells, respectively (first row, horizontal colored arrows in Fig. 2). However, and quite unexpectedly, neurons belonging to the non-adapted site B (located 4° away) also shifted their respective optimal orientation tuning curves. The orientation tuning curve of the orange neuron shifted in the repulsive direction (68.9°), while red and purple units displaced their respective optimal peaks towards the adapter (49.3° and 86.1° for red and purple neurons, respectively, middle row). Cells belonging to the third non-adapted site C (bottom row) also shifted their respective orientation tuning curves (22.1° for sky neuron, 38.3° for green neuron, and 27.4° for gray neuron) in the attractive direction, that is, the novel orientation approached the adapter. It is worthwhile to emphasize that, at this site the amplitude of the shift is smaller perhaps due to the larger distance separating both receptive fields (A and C, see Fig. 2 insert). Thus, in spite of the fact that cells excited within RF's B and C were not adapted, they reacted by showing new preferred orientations. Furthermore, the response magnitude of the novel optimal orientation was about equal to the strength of the original preferred orientation. Other examples are shown in a supplementary figure (Fig. S2).

### Receptive-fields separation

The excitatory receptive field dimensions of the multi-unit activity extended from 2.5° to 4° (average = 3.8 ± 0.4°). All RF's were centrally located within a 15° radius from fovea (Fig. 3a). The adapting stimulus had equal dimensions and covered the excitatory receptive area. Measurements of receptive field size in area 17 as a function of eccentricity indicate that within a radius of 15° of fovea, RF dimensions rarely extend 5°^18^,^19^. The average distance separating the adapted and non-adapted fields was 8.0 ± 3.0° (histogram distribution shown in Fig. 3b, the downward triangle indicates the mean value). Most significantly, the absence of cross-influences between units excited through distant receptive fields was tested by measurement of spontaneous activity because the latter is weak and it readily fluctuates if a stimulus encroaches on the periphery of the companion receptive field. An example is displayed in Fig. 3c, indicating the levels of spontaneous firing while at the same time when the distant companion RF is stimulated with the selected range of orientations. In this example the distance separating both receptive fields was 6°. The spontaneous firing remained unchanged for all applied orientations (X-axis) and for every trial (a single dot stands for one trial presentation of the sine-wave grating applied for 4.1 s; one-way ANOVA: p > 0.05, Shapiro-Wilk normality test: w = 0.9, Bartlett's test for variances: p = 0.8). The average firing at spontaneous activity in control conditions was 15.8 Hz and in adaptation 15.76 Hz (t-test: p > 0.05, Shapiro-Wilk normality test: w = 0.9, F = 1.2). It must also be emphasized that RF's were never stimulated concurrently (see methods). Thus, RF's were sufficiently far apart in order to avoid overlapping surrounds. In addition, we observed non-significant differences of spontaneous activity across all the presented orientations within all RF's when stimulating the adjacent RF's pre- and post-adaptation (one-way ANOVA: p > 0.05, Shapiro-Wilk normality test: w = 0.8, Bartlett's test for variances: p = 0.8, Fig. 3d). Another example is illustrated in Fig. 3e. It shows the spiking activity of neurons from one RF (RF A, blue, *i.e.*, non-stimulated) during the stimulation of the adjacent RF (RF B, gray), and during the stimulation of the RF itself (RF A). Conversely, the activity was computed in a similar fashion for RF B when RF A was stimulated, and when RF B was stimulated.

We observed a significant difference between these two conditions for both RF's (unpaired t-test, p < 0.05, Shapiro-Wilk normality test: w = 0.9, F = 1.7, Fig. 3e). Furthermore, stimulating one RF did not modify the firing at spontaneous activity (red arrow, Fig. 3e). This is highly incompatible, had we encroached the boundaries of the companion RF.

### Preferred orientation stability

**Figure 4:**
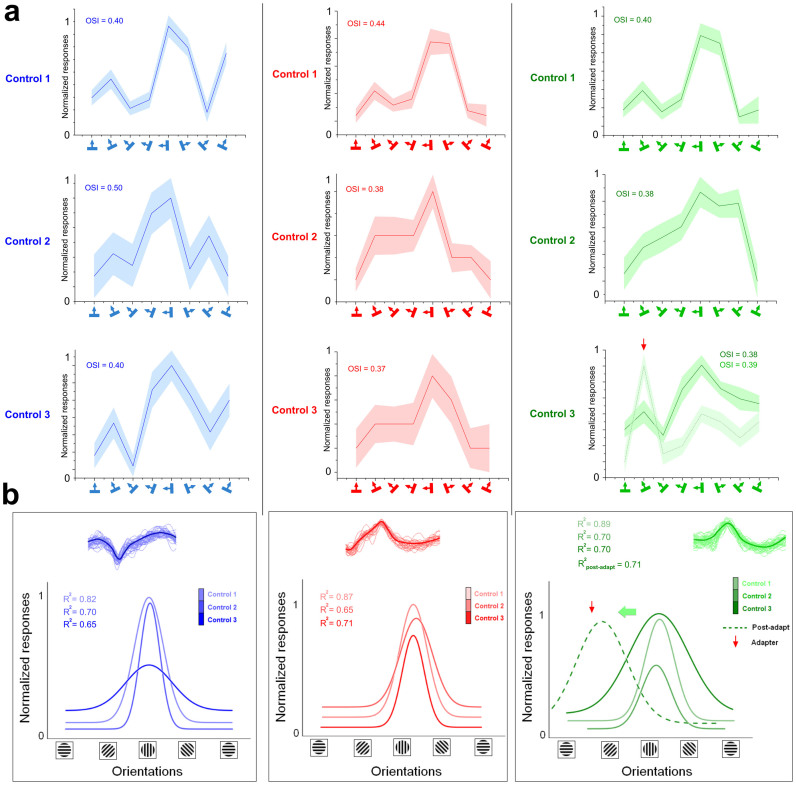
Tuning stability. (a) Raw tuning curves of three neurons at different time-windows (90 min between control 1, control 2, and control 3). As an additional example, the green cell (right) shows the post-adaptation tuning curve (dashed tuning curve, red arrow head corresponds to the adapting orientation) demonstrating the classical observed tuning shift (horizontal green arrow); Y-axis is the normalized amplitude of the cell's firing. (b) Gaussian fits derived from raw data. Spike-waveforms for every neuron are shown above the Gaussian curves.

It is important to confirm that optimal orientations do not fluctuate spontaneously. Many investigations have shown that for any given cell, although response magnitude varies, the preferred orientation remains remarkably constant for several days^3^,^20^,^21^. Nevertheless, we proceeded with an additional control by measuring orientation tuning curves of three cells during a four hour period (about 90 min separated each recording session, Fig. 4). Raw responses are shown in Fig. 4a and Gaussian fits for each cell are illustrated in Fig. 4b. While the evoked firing rates vary in amplitude, the optimal response (normalized) is elicited by the same orientation in all three recorded neurons. Following these retests, adaptation (12 minutes) was carried out that resulted in a shift of the peak of the tuning curve (see example in Fig. 4, right, the green cell shifted by 45° in the attractive direction).

### Shifts in non-adapted sites

**Figure 5:**
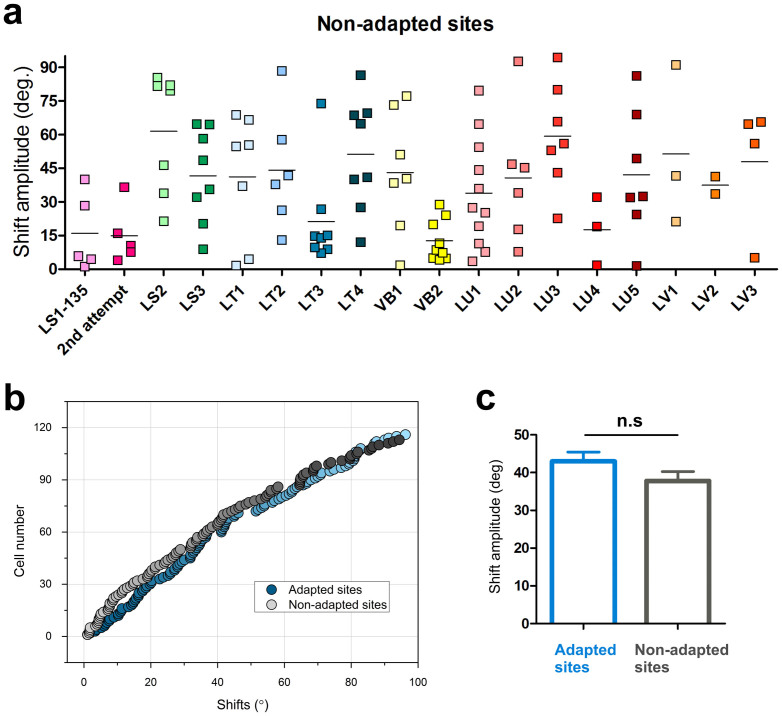
General survey. (a) Shift-magnitude of every neuron in every cluster; Y-axis: shift amplitude, horizontal line: mean-shift for the corresponding cluster. Notice that the recording “LS1 135” is repeated (second attempt). X-axis: cluster code. For sake of clarity, cells recorded from closely positioned electrodes are grouped within one cluster. (b) Cumulative plots of orientation shifts, magnitudes in increasing order; the close parallelism between both curves suggests that in adapted (blue) and non-adapted (gray) cells, the shifts are comparable. (c) The equal average magnitude shifts confirm that there is no difference between adapted and non-adapted sites.

Figure 5a illustrates the range of orientation shifts on a cell to cell basis (the short horizontal line indicates the average shifts for a particular cluster of cells). Clusters, *i.e.*, cells recorded from the same site, are identified on the X-axis and the Y-axis indicates shift-range of every unit belonging to non-adapted neurons. In total, 108 units were analyzed. Notice that the first two groups of neurons (labeled “LS1-135” and “Second attempt”) are same units tested twice in order to ascertain the consistency of shifts in cells at the non-adapted locus. In this example, the mean magnitudes of shifts were 19.7° (first attempt) and 15.6° (second attempt). Therefore, it can be deduced that orientation shifts at non-adapted sites are the consequence of adaptation executed at some distance from the tested location. Although the average shift-magnitudes were about equal, it should be emphasized that within a single cluster (cells likely to be physically close to each other) some units significantly shifted their preferred orientation, whereas other neighboring cells failed to change their preferred orientation, hence suggesting that orientation changes are specific characteristics belonging to some neurons (cluster LU3 for instance). This last result indicates that changes of orientation may not be attributed to a general surge of excitation (see below). These results corroborate recent reports showing that within a large population of neurons in sensory cortices, there are embedded sub-networks of units whose selectivity is modifiable depending on a particular stimulus protocol^22^. Such a manifold potential for modification depending on groups of particular cells leads to the conclusion that within a pool of neurons, a class of cells maintains its initial property (original orientation); while another group of intermingled units has a potential to change its optimal stimulating feature to a newly acquired orientation. This heterogeneous organization echoed by dissimilar shifts following adaptation could allow the preservation of an overall stable network yet allowing adaptation to novel conditions of the visual environment.

In Fig. 5b, absolute orientation shifts' magnitudes are plotted in increasing order for every tested cell (cells are not paired, adapted site = 110; non-adapted site = 108). The very similar profiles of cumulative-type curves of shifts in adapted and non-adapted sites are indicative that in both sites the peaks of the orientation tuning curves are displaced with equal magnitude. Although the change of orientation in individual cells may vary, the overall mean shift-amplitude is about equal in adapted and non-adapted cells: 42.9° and 37.7°, respectively (Fig. 5c, t-test: p > 0.05, Shapiro-Wilk normality test: w = 0.9, F = 1). These data suggest that when a group of neurons modifies its orientation selectivity, some other cells follow the change in an equivalent magnitude. Analogously, it is like the domino effect: once the first block falls the other blocks tilt and fall accordingly. That is, a full set of orientation axes is represented with no orientation-hole. Alternatively, if such collective shifts were deficient, one orientation (shifted) would be represented twice since it would closely match an already present axis. Such double presentation of one axis of orientation appears incompatible with the notion that a single hypercolumn contains full range of orientations.

The above results along with data reported in the literature suggest that, following adaptation, the original equilibrium between multiple synaptic drives is ruptured. Consequently a novel optimal orientation arises^9^,^23^. In addition, we observed significant changes of another parameter: the tuning bandwidth. Indeed, the overall bandwidth at half magnitude is significantly narrower after adaptation for both sites (Control: 32.1 ± 1.7°; post-adaptation: 26.9 ± 1.4°, unpaired t-test: p < 0.05, Shapiro-Wilk normality test: w = 0.8, F = 1.3). This narrowing of the bandwidth following adaptation is coherent with the slight augmentation of the OSI after adaptation as described above.

### Relationships between magnitude of shifts and inter-RF distance and orientation differences

**Figure 6:**
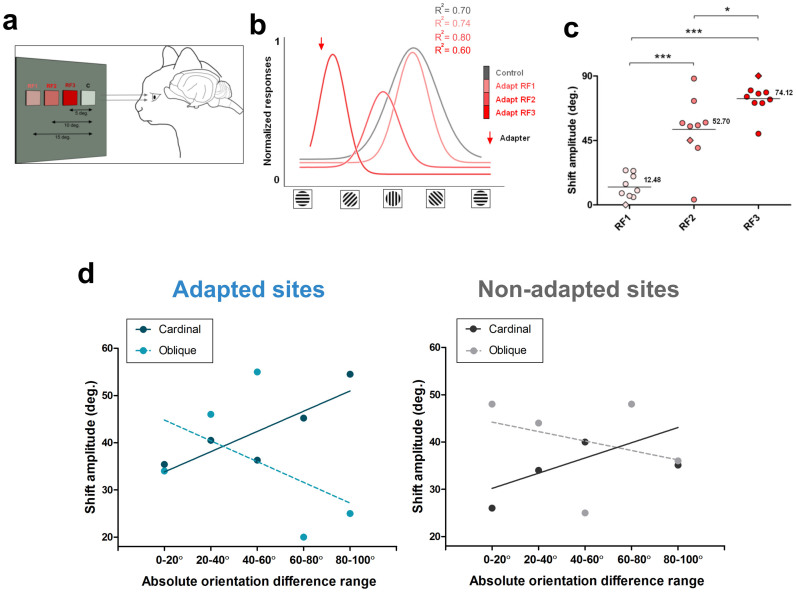
Inter-receptive-field distances. (a) Experimental set up of stimulation and visual adaptation in relation to inter-RF distances. (b) Typical example of tuning curves of one neuron, responses normalized. The adapting site was approached in steps of 5° from 15° (RF1) — the largest distance separating both fields to the closest gap: 5° (RF3). The example shows that as the adapting field gets closer to the control receptive field, the stimulated neurons elicited larger shifts of the orientation tuning curve towards the adapter (downward red arrow). (c) Global distribution of the averaged shift-magnitudes in relation to inter-RF distances (same cells in all conditions). (d) Shift-amplitudes in adapted and non-adapted sites in relation to the original preferred orientation prior to adaptation, the broken line represents cells whose initial preferred orientation was oblique, while the solid line stands for units whose initial preferred orientations were cardinal (vertical or horizontal, see text for details). X-axis: absolute orientation differences between preferred orientation and the adapting orientation.

We then examined the spatial extension of adaptation. We measured the amplitude of shifts in relation to the distance separating the two stimulated loci measured from center to center (Fig. 6a–c). The adapting grating was applied in steps at three distances: 5°, 10°, and 15° from the tested site (Fig. 6a, tested site identified by letter C). When both RF's were separated by 15°, the tuning curves of neurons were not modified in strong fashion, although the shifts were significant (average shift = 12.48°). Then, as the adapted field approached the tested field to 10° (RF2), the neurons shifted their optimal orientation with increased amplitude in the attractive direction (average shift = 52.70°). At the third adapted site (RF3) located 5° off the tested field (RF C), neurons showed an even larger shift, (average shift = 74.12°) (Fig. 6b, c). Figure 6c shows the distribution of shift amplitudes over a neuronal population (n = 9). It demonstrates the relationships between the distance separating both RF's and the shift amplitude (Shapiro-Wilk normality test: w = 0.8, t-test RF1-RF2, p < 0.05; t-test RF2-RF3, p < 0.05; t-test RF1-RF3, p < 0.05). Thus changes of orientation selectivity spread over a distance of up to 15°.

Previously^9^ it has been reported that in adapted receptive fields cells tuned to cardinal orientations have larger shift-amplitudes when the orientation difference between the adapter and the original orientation increased. Conversely, cells tuned to oblique orientations exhibited larger shift amplitudes when the orientation difference was small, and shifts decreased as this gap increased. Figure 6d illustrates the shifts magnitudes between adapted and non-adapted sites when cells were grouped in relation to their initial optimal orientation. Thus cells with original cardinal orientations: 90° ± 22° and 0° ± 22° (solid line) were dissociated from cells with initial oblique orientation, 45° ± 22° (broken line). Furthermore, for these analyses, neurons were grouped into five 20° orientation classes (Fig. 6d). Interestingly, the same relationships were observed in both adapted and non-adapted sites. These data could signify that in order to maintain constancy of orientation organization following the shifts in the adapted and non-adapted sites, the cortical network rectifies its original orientation layout to maintain its orientation selectivity distribution. In other words, a group of neurons in area 17 displaces the peaks of its orientation tuning curve in relation to the orientation of the neighboring neurons as reference axis.

## Discussion

**Figure 7:**
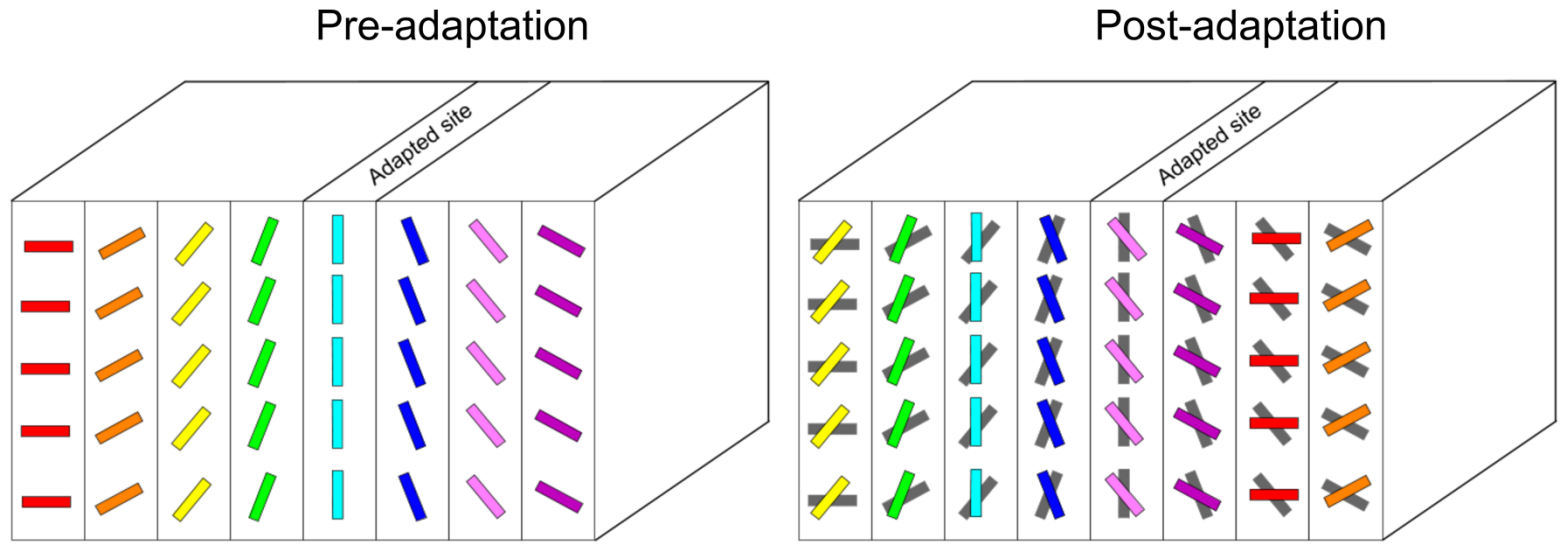
Schematic model illustrating cortical reorganization following visual adaptation. “Ice cube” model exhibiting orientation columns. After adaptation, a tilt of orientation axis is displayed. Notice an absence of orientation hole.

The results demonstrate that preferred orientation of neurons in the visual cortex changes following a relatively short period (12 min) of localized adaptation taking place remotely from the tested (non-adapted) receptive field. Data from our experiments have the following implications: (1) selectivity of orientation organization in area 17 is not attributed to a determined anatomically based and/or ontogenetic layout of a neuronal network. Rather, orientation column design appears to be a labile formation changing its selectivity depending on the history and more generally stimuli conditions exciting neighboring neurons; in other words, local persistent stimulus leads to unmapping and remapping of cortical orientation domains (Fig. 7). Thus, a cross-mutual influence may redraw what has been thought to be an immutable organization of the orientation layout. (2) It appears that orientation columns are a functional construction rather than strictly anatomical, one based on parallel segregation of input fibers emanating from the retina, and then from the lateral geniculate nucleus (LGN). These results may appear astonishing since orientation columns in the visual system are thought to be rather stable after the critical period that follows birth because coding visual trigger features necessitates highly discriminatory neuronal properties^24^.

### Methodological considerations

It may be argued that shifts in orientation are the consequences of spontaneous fluctuations of levels of firing rates. Although such a hypothesis may not be rejected, nonetheless, numerous experimental results argue against this eventuality. Recently, it has been shown that orientation selectivity in identified units remains stable over several days^20^. In addition, it is unlikely that response-changes may be ascribed to random fluctuations of cellular excitability. Several authors have demonstrated that orientation selectivity is invariant^3^, as the jitter of the optimal orientation is small (<5°). Finally, the response modulations of cells in the adapted and non-adapted sites are constrained roughly to the adapter and the initial preferred orientations. For instance, evoked responses close to the adapter are augmented while, in parallel, responses to the original preferred orientation are weakened^9^; such dual modulations in opposite directions cannot be reconciled with global fluctuations of excitability. Collectively, all of these arguments indicate that it is very unlikely that the described specific discharge modulations are due to spontaneous surges of excitability. Moreover, anesthesia eliminates the impact of attention. Imposing an orientation to a particular neuron for twelve minutes while the animal is paralyzed and anaesthetized is a situation that clearly does not replicate natural conditions. This is a compromise needed to induce neurons to change their preferred orientation. In many reports, it has been shown that imposing a sensory or appropriately timed electrical stimulus induces neuronal property changes within minutes^25^,^26^,^27^,^28^,^29^, which is a time scale congruent with adaptation duration of the described experiments. Although the change in preferred orientation may last for many minutes^30^ and may be ten times longer than adaptation duration^7^, data suggest that the brain's network is dynamic as it is attuned to the environmental conditions. This may imply that functional column changes are transient yet lasting for many minutes.

Twelve minutes of continuous adaptation may raise the question of the duration of recovery. Literature reports that even after two hours following adaptation; only half of the neurons recover their original selectivity^7^,^27^,^30^. Indeed, while capturing the activity of both sites (adapted and non-adapted) post-adaptation; we started first with the adapted site by presenting it the grating corresponding to the original optimal orientation, thereafter, showing the grating corresponding to the original optimal of non-adapted site. In a systematic fashion, after the presentation of these gratings corresponding to the original optimal controls in both sites, we presented the adapter-grating alternatively to both sites (adapted then non-adapted). As evident from our data that most of the neurons exhibited shifts of the tuning curves superior to 10 degrees (both sites) post-adaptation (90% from the adapted sites, 80% from the non-adapted sites, Fig. 5a), thus indicating that they are indeed the result of the adaptation process.

**Figure 8:**
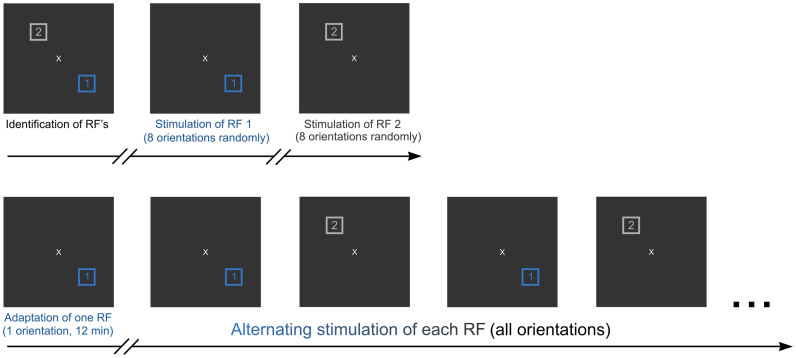
Visual stimulation and adaptation protocols. Upper part: initial stimulation (control). Lower part: adaptation process and post-adaptation recordings.

Another methodological aspect may be clarified. In order to avoid surround cross-influence between the receptive fields, neurons stimulated by each site were never excited simultaneously (see Fig. 8). In this study, the average distance separating the adapted and non-adapted fields was 8.0 ± 3.0°. It has been shown that far-surround radius in V1 averaged 5.5 ± 2.64°^31^. This reduces the probability of extensive overlapping of both RF's. To our knowledge, no study has demonstrated that stimulating the surround of RF affects the orientation selectivity of neurons. Rather, the stimulation of the periphery results in the modulation of the firing rate (see Fig. 6b). Furthermore, as mentioned above, the spontaneous activity of each RF was never affected by the stimulation of the companion RF.

### Mechanisms

Our results are supported by several recent reports. In mouse it has been demonstrated that a single dendritic branch exhibits, over a short distance, synaptic inputs with a large spectrum of orientation channels allowing mutual influences^32^. Whole-cell recording in rodent V1 shows large sub-threshold depolarization evoked by non-preferred orientations^33^,^34^,^35^. After adaptation, the impact of an applied orientation takes over the cell's excitability causing the emergence of a novel optimal orientation due to a specific excitatory drive, wherein one input dominates over other inputs, thus, driving the synaptic strength above threshold that provides the cell its orientation selectivity. Recently in human, it was demonstrated that when the neuronal processing is disturbed, another cortical area takes over the task to maintain subject performance^36^. These investigations, although at different levels, suggest that single cells or cortical areas may modify their destined commitment to specific functions or tasks following changing conditions, and thus shift the responses to new stimulating environments^37^. The above assumptions are supported by recent reports describing “classes of neurons that accumulate information from an entire cortical column and broadcasts outputs to distant targets”^22^.

Neurons in the primary visual cortex receive polysynaptic inputs not only from neighboring cells, but also from a network of distal cortical sites. Indeed, feed-forward and feedback connections are extensive. Thus, area 17 neurons' preferred orientations are not exclusively resulting from feed-forward inputs originating from aligned lateral geniculate nucleus cells^2^, since elicited responses are also influenced by long-range connections^38^,^39^. In particular, the cat's secondary visual cortex (area 18) exhibits strong reciprocal relations with area 17^40^, which relays parvocellular inputs to area 18. Monier et al.^25^ demonstrated by recording membrane potentials intracellularly that a single cortical cell is receiving inputs from a large variety of synaptic inputs. They came to the conclusion that the diversity of input combinations may be reflected in homogeneities of intra-cortical patterns of connectivity leading to changeable properties. It is to be expected that once the adaptation modifies the balance between inputs and specifically the gradient between excitatory and inhibitory synaptic weights, the neurons become susceptible to exhibit a novel optimal orientation. Because of the extensive intra-cortical network a particular neuron that modifies its selectivity influences other cells with which it is directly or indirectly connected, thus contributing to modifications of the orientation selectivity of an entire network (Fig. 7). Such neuronal dynamics have been described in the auditory cortex^41^.

In conclusion, our results strongly suggest that orientation selectivity is a rapidly modifiable characteristic that is adjusted by specific neurons depending on the behavior of cells surrounding the tested neurons. Thus, orientation columns transcend anatomy, and are functionally dynamic entities.

## Methods

### Ethical approval

Animal surgery and electrophysiological procedures followed the guidelines of the Canadian Council on Animal Care and were approved by the Institutional Animal Care and Use Committee of the University of Montreal. Animals were supplied by the Division of Animal Resources of the University of Montreal.

### Animal preparation

Electrophysiological recordings were performed using multielectrodes within area 17 of adult domestic cats. Adult cats (2.5–3.5 kg, age 12–24 months) of either sex, sedated with acepromazine maleate (Atravet, Wyeth-Ayerst, Guelph, ON, Canada; 1 mg kg^−1^, intramuscular) and atropine sulfate (ATRO-SA, Rafter, Calgary, AB, Canada; 0.04 mg kg^−1^, intramuscular), were anaesthetized with ketamine hydrochloride (Rogarsetic, Pfizer, Kirkland, QC, Canada; 25 mg kg^−1^, intramuscular) maintained with 0.3% isoflurane (AErrane, Baxter, Toronto, ON, Canada). Lidocaine hydrochloride (Xylocaine, AstraZeneca, Missis-sauga, ON, Canada; 2%) was injected subcutaneously as a local anaesthetic during surgery. A tracheotomy was performed for artificial ventilation, and one forelimb vein was cannulated to administer a nutritive solution containing a paralyzing agent. Animals were then placed in a stereotaxic apparatus. Xylocaine gel (Astra Pharma, Mississauga, ON, Canada; 5%) was applied on the pressure points. For the remaining preparations and recording, paralysis was induced with 40 mg and maintained with 10 mg kg^−1^ h^−1^ gallaminetriethiodide (Flaxedil, Sigma Chemical, St. Louis, MO, USA; intravenous) administered in 5% dextrose lactated Ringer's nutritive solution. General anaesthesia was maintained by artificial ventilation with a mixture of N_2_O/O_2_ (70:30) supplemented with 0.5% isoflurane (AErrane, Baxter, Toronto, ON, Canada) for the duration of the experiment. Proper depth of anaesthesia was ensured throughout the experiment by monitoring the EEG, the electrocardiogram and expired CO_2_. In addition the heart rate remained unmodified after skin stimulation. The end-tidal CO_2_ partial pressure was kept constant between 25 and 30 mmHg. A heating pad was used to maintain a body temperature of 37.5°C. Tribrissen (Schering-Plough, Pointe-Claire, QC, Canada; 30 mg kg^−1^ per day, subcutaneous) and Duplocillin (Intervet, Withby, ON, Canada; 0.1 ml kg^−1^, intramuscular) were administered to the animals to prevent bacterial infection. The pupils were dilated with atropine sulfate (Isopto-Atropine, Alcon, Mississauga, ON, Canada; 1%) and the nictitating membranes were retracted with phenylephrine hydrochloride (Mydfrin, Alcon, Mississauga, ON, Canada; 2.5%). The loci of the area centrales were inferred from the positions of the blind spots, which were ophthalmoscopically focused and back projected onto a translucent screen. Plano contact lenses with artificial pupils (5 mm diameter) were placed on the cat's eyes to prevent the cornea from drying (University of Montréal, PQ, Canada).

At the end of each experiment, euthanasia was achieved with a lethal dose of pentobarbital sodium (Somnotol, MTC Pharmaceuticals, Cambridge, ON, Canada; 100 mg kg^−1^) by intravenous injection.

### Electrophysiology and visual stimulation

Neurons from several recording sites in layers 2/3 were simultaneously recorded. Electrodes were lowered either tangentially (with a 20–30° angle, tetrode arrangements) or vertically (four electrodes, inter-electrode separation: 400 μm, Frederick Haer & Co, Bowdoinham, ME, USA; 2–10 MΩ at 1 kHz). The signal from the microelectrodes was amplified, band-pass filtered (300 Hz–3 kHz), digitized, and recorded with a 0.05 ms temporal resolution (Spike2, CED, Cambridge, England). In all cases at least two excitatory receptive fields were outlined. We paid particular consideration that surrounds of receptive fields were not overlapping.

Firstly, in order to minimize direct cross-influence between both sites, respective cells were never stimulated at the same time (Fig. 8). Secondly, the spontaneous spiking activity of each neuronal population belonging to one RF was recorded during the stimulation of the adjacent RF (see results). Finally, the surround influence in responses evoked from excitatory area could modulate the magnitude of responses but the neuronal selectivity remained unaffected (see Fig. 6b).

Each site was stimulated with drifting sine-wave grating patches covering the excitatory fields (Spatial Frequency = 0.24 cycle/°, Temporal frequency = 1.0–2.0 Hz, Contrast = 80%). Cells were sequentially stimulated in pseudorandom presentation of gratings. The grating was placed in the center of the aggregate RF of the sampled units. We measured tuning with eight equally spaced orientations (22.5° interval) of the drifting sinusoidal grating. It is to be underlined that the gratings were presented only for one direction. Stimuli were presented in each receptive field in isolation, thus each group of cells was stimulated alternatively in order to eliminate direct cross influence between neurons (Fig. 8). Adapting and stimulating gratings were identical in size and characteristics. Prior to adaptation, respective tuning curves were obtained revealing the preferred orientation in each site that elicited the maximal firing rates. In the following step, only one site was adapted by applying a non-preferred orientation up to 90° off the initial preferred orientation of the neurons (usually evoking a weak response) continuously for twelve minutes. While the adaptation happened over one site, the second site was unstimulated. After adaptation, orientation tuning curves were re-investigated in the same manner as before adaptation.

### Cell isolation

Given that the multi-unit activity was recorded concurrently from the same tips using multi-electrodes, it was essential to ascertain that cells were well isolated, because the same unit may exhibit sufficiently different waveforms (for instance, magnitude in relation to distance between recording tip), and thus belong to different clusters in principal component analysis.

Cell-separation was based on spike-waveforms, cluster-isolation using first principal components analyses, autocorrelograms, and trial-count correlation (TC). No more than 5 cells were recorded from the same electrode tip. TC denotes the trial by trial Pearson correlation-coefficient between simultaneously recorded firing of two neurons in response to the presentation of an identical stimulus. In response to the presentation of the same stimulus, the same unit fires identically, regardless of spike amplitude. In order to eliminate such occurrences we correlated neural activities of every cell-pair for every applied trial (25 trials, same stimulus, duration 4.1 s). Since optimal orientations eliciting maximal firing rates were chosen for this computation, and considering a relatively long time-window of analyses (4.1 s), we should expect a high value for correlation if it were the same unit, because such time windows of analyses are sufficiently large to capture the full strength of correlation. Indeed, correlations are underestimated if the counting window is too short. In all analyzed cells the TC was extremely weak (TC < 0.3). Such extremely feeble TC establishes that both spike trains do not originate from the same neuron (see Supplementary Fig. S1). Autocorrelograms preclude contamination by spikes of other units. Anesthesia and paralysis of animals reduced the possibility of similar modulations of firing patterns in cells of the same clusters due to rapid eye motion or attention.

### Data analysis

Once single cells were sorted out off-line from multi-unit spike trains accumulated during data acquisition, Gaussian tuning curves were constructed from raw data. We fitted our raw data with the Gaussian function to determine with precision the preferred orientation of neurons and then measured shifts in orientation preference. We used the following Gaussian function:
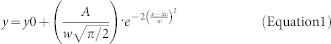
where y0 is the offset, Xc is the center, w is the width, and A represents the area under the Gaussian fit.

The shifts of peaks of tuning curves between pre- and post-adaptation conditions were calculated from the Gaussian fits using the following formula:
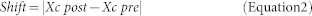
where Xc is the central value derived from the Gaussian fit.

Orientation selectivity index (OSI) was calculated from data as the difference between firing rate (FR) at preferred and orthogonal orientations as follows:
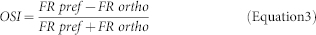
The closer the OSI is to one, the stronger the orientation selectivity (see results).

It has been shown previously that averaged shifts inferior to 5° were not significant^9^, however we consider all the shifts in the present investigation.

One-way ANOVA (95% confidence limit) statistical tests were used to compare the spontaneous firing activity between all presented stimuli. Statistical comparisons were performed using unpaired sample two-tailed t-test (95% confidence limit) for unpaired data. Shapiro-Wilk normality test (significance threshold = 0.05) was used for the normal distribution of data, and Fisher test to compare variances for all student tests.

Bartlett's test was performed to compare variances for ANOVA tests.

## Author Contributions

L.B., V.B. and S.C. contributed equally to this work. S.M. designed the study, contributed to data analyses and wrote the manuscript. J.R. contributed to data analyses.

## Supplementary Material

Supplementary Information Supplementary figures
