# Supplementary material for: Reprogramming of orientation columns in visual cortex: a domino effect
Source: Sci Rep. 2015 Mar 24;5:9436. doi: 10.1038/srep09436 (PMC4371149; doi:10.1038/srep09436)
Supplement: Supplementary Information — Supplementary figures [file srep09436-s1.pdf]

Reprogramming of orientation columns in visual cortex: a domino effect

Lyes Bachatene, Vishal Bharmauria, Sarah Cattan, Jean Rouat, Stéphane Molotchnikoff

Supplementary material:

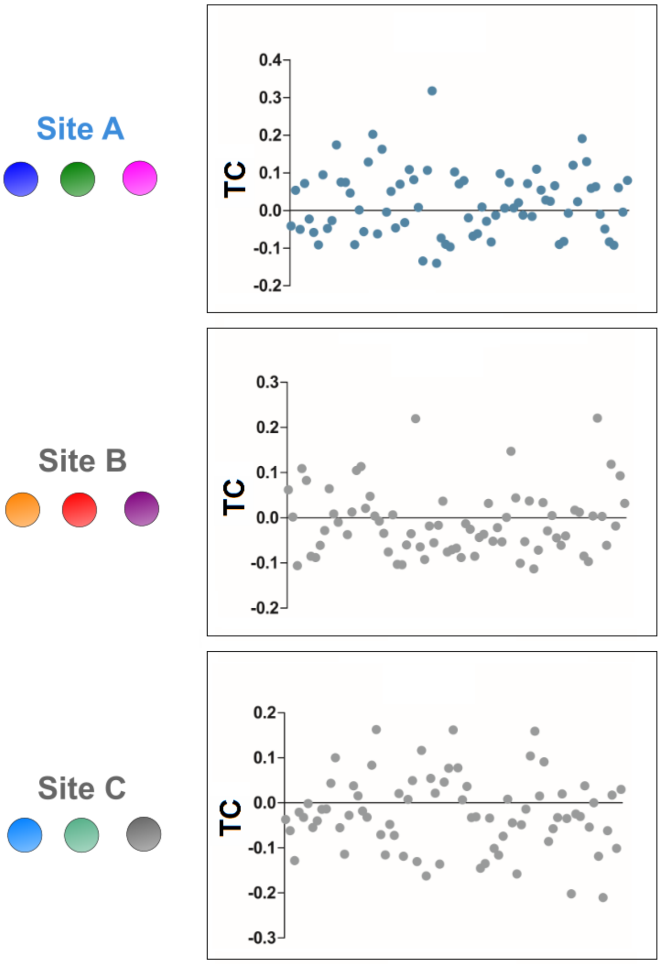

Figure S1 Cell's isolation. Scatter plots indicate Pearson correlation (TC) of spike-counts for every stimulus trial (n = 25) between all cells of each site. Each data point indicates the value of the Pearson correlation for every trial (X scale arbitrary). The extremely low values (< 0.3) ascertain that spikes originate from different neurons.

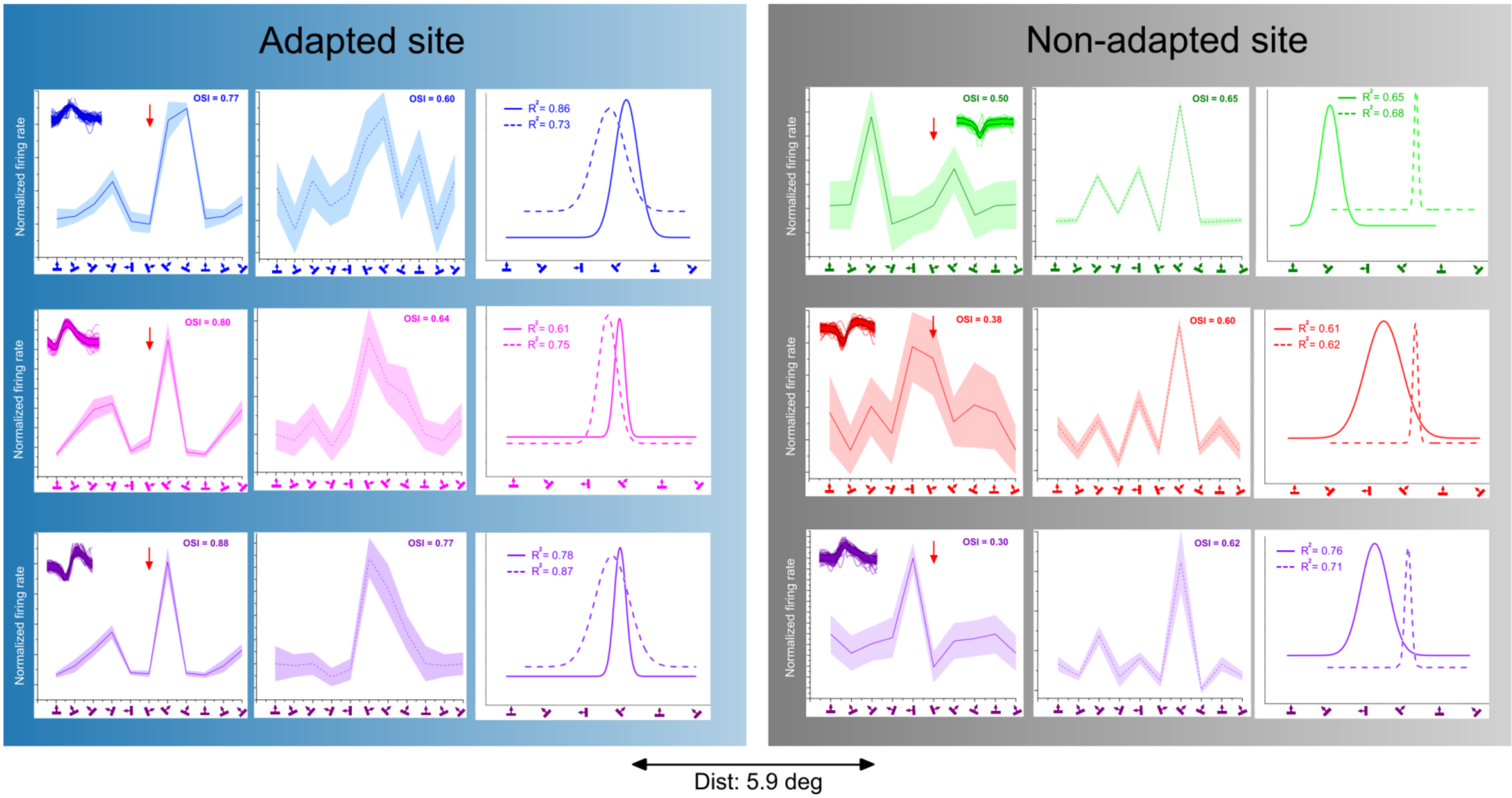

Figure S2 Additional examples. Left part shows the adapted site and right part represents the non-adapted site. The distance separating both RF's was 5.9 deg. Spike-waveforms are shown within the raw data boxes. On the right of each raw data are illustrated the Gaussian fits for every neuron. The downward red arrow indicates the adapting orientation. Light colors represent error bars.
